# Intervention of RT-ABCDEF for cancer

**DOI:** 10.3325/cmj.2019.60.55

**Published:** 2019-02

**Authors:** Chun-Song Hu

**Affiliations:** Nanchang University, Jiangxi Academy of Medical Science, Hospital of Nanchang University, Nanchang, China *cnhucs@163.com*

New phase-III clinical trials showed that combining immune checkpoint inhibitors – atezolizumab (1), pembrolizumab (2), or durvalumab (3) – with cytotoxic chemotherapy improves the treatment efficacy in patients with different types of lung cancer. These trials therefore confirm that enhancing tumor-specific T-cell immunity by inhibiting programmed death-ligand 1 or programmed death-1 signaling is promising in the treatment of lung cancer, which is one of the most frequent cancer types.

In the United States, over 1.7 million new cancer cases and more than 600 000 cancer deaths were reported in 2018 (4). During the past decade, the incidence rate in women was stable and in men it declined by approximately 2% annually, while the death rate declined by about 1.5% annually (4). Similar patterns were observed in Croatia (5). These substantial achievements can be attributed to the development of new technologies and anti-cancer agents, as well as early diagnosis, advanced treatment, and overall prevention. However, there are still large inequalities, which may be linked not only to limited access to high-quality health care but also to unhealthy lifestyle. Potentially modifiable risk factors, including chronic diseases, are now confirmed to highly associate with cancer risk (6,7). Of course, there are also non-modifiable risk factors, such as sex, age, and family history.

Strong evidence shows that cancer incidence can be decreased by adhering to a healthy lifestyle. Higher coffee consumption was associated with a lower risk of death from various causes (8,9), whereas hot tea consumption was associated with an increased risk for esophageal cancer (10), especially when combined with related risk factors, such as excessive alcohol or tobacco use. Other behavioral risk factors include housing and food insecurity, e-cigarettes use, consumption of ultra-processed foods, toxins exposure, health care access, and health status.

Current status of cancer in the world calls for new strategies and measures for cancer control and prevention. The author of this text previously developed one such strategy (11), ie, intervention of RT-ABCDEF (iRT-ABCDEF), meaning “follow-up, examination, disease and risk factor control, changing unhealthy lifestyle and cutting spreading pathways, biohazard control, and antagonistic treatment” as an intervention of routine, right, and reversible treatment (iRT) (Table 1). Similar to the magic and novel “polypill” (SEEDi^1.5^) (12), which is based on five core healthy elements “environment-sleep-emotion-exercise-diet,” it is an effective strategy for prevention and control of major non-communicable diseases (mNCDs), suitable not only for CVD but also for cancer and diabetes. For example, physical activity is a simple way to reduce the cancer risk associated with chronic diseases (7). In fact, many chronic diseases result from unhealthy lifestyle, such as exposure to air pollution, noises, staying up late, long term depression, lack of physical activity or obesity, heavy drinking or smoking, and diets with high salt, sugar, and lipid intake and low vegetables and fruits intake. Both basic and clinical studies in recent years have shown that associations among diet, gut microbiome, and human immunity lead to cancer and affect anticancer treatment (13,14).

**Table 1:** iRT-ABCDEF as a standardized comprehensive program for cancer

iRT-ABCDEF	Tips
F	**Follow-up**, especially of patients with family history, precancerous lesions, and unhealthy lifestyle for primary and secondary prevention, tracking outcome, and evaluating drug, surgical, or radiotherapy effects. It is very important for large-scale epidemiology investigations.
E	**Examination** for early diagnosis, treatment, and primary and secondary prevention, which includes large-scale screening and regular comprehensive or targeted physical examination, such as testing for biomarkers (eg, AFP, CEA, PSA, CA 125, CA 19-9, CA 15-3) and genetic variants (eg, *BRCA1* and *BRCA2*), gastroscopy, colonoscopy, molybdenum photography, echocardiography, biopsy, immunohistochemical or histopathologic analysis, computed tomography and single photon emission computed tomography, and magnetic resonance imaging.
D	**Disease and risk factor control**, including family history, diabetes, chronic infection (HP, CMV, EBV, HPV), chronic hepatitis, gastritis or ulcer, intestinal or gallbladder polyps, heavy drinking or smoking, physical inactivity, and obesity.
C	**Change unhealthy lifestyle and cut spreading pathways** by SEEDi^1.5^ or SEEDi^3.0†^ strategies, such as cessation of smoking or drinking, and cutting spreading or genetic pathways by isolation of patients, and gene knockout or gene editing technologies.
B	**Biohazard control**, including control of abnormal symptoms and signs, abnormal physiological indexes and biomarker level, precancerous lesions, family history, unhealthy lifestyle, high-risk occupation, and early tumor metastasis and recurrence. Grade-zero prevention based on “Health in All Policies and Laws” is a good choice.
A	**Antagonistic treatment**, including chemical agents, such as statin-based treatment, precision radiotherapy, immunotherapy with checkpoint inhibition, cancer vaccine, and other new methods. Sometimes, traditional Chinese medicine is also a good choice for cancer treatment.
iRT	**Intervention** with above strategies as **routine, right, and reversible treatment**

*AFP – alpha-fetoprotein; CEA – carcinoembryonic antigen; PSA – prostate-specific antigen; CA – cancer antigen; HP – *Helicobacter pylori*; CMV – cytomegalovirus; EBV – Epstein-Barr virus; HPV – human papillomavirus.

†SEEDi^1.5^ strategies were developed based on five core healthy elements, that is, external and internal environment, sleep, emotion, exercise, and diet intervention [E(e)SEEDi]. When E(e)SEED-BasED lifestyle is combined with iRT-ABCDEF and Grade 210 prevention, it is 3.0 version of SEEDi strategy (SEEDi^3.0^).

Measures such as smoking ban and laws on air pollution are important because grade-zero prevention plays a key role in fighting cancer. It is time to enhance public knowledge not only on “Health in All Policies” but also “Health in All Laws.” New strategies will help countries worldwide make a breakthrough in cancer management and prevention. One of such strategies is iRT-ABCDEF, a fair, accessible, and independent technology, which can easily achieve universal health coverage.

Decreases in overall breast cancer mortality from 2000 to 2012 were associated with advances in screening and adjuvant therapy (15). Survival trends are generally increasing, but differences among countries remain wide, especially for cancer in children (16). New strategies or technologies for reducing cancer morbidity and mortality have to be developed, for example, new biomarkers, such as lymphocyte phenotypes, that could predict checkpoint inhibitor response, as well as reagent kits for early diagnosis of cancer, vaccine, and stem cell, gene, or base-editing technology for patients with a family history of cancer. There are also biomarkers that still need to be confirmed for screening and therapy-making decisions (17). Cancer immunotherapy of checkpoint blockage has been a breakthrough. However, it is time to conduct iRT-ABCDEF as a new standardized comprehensive program for better management or self-management of cancer in countries worldwide.
